# The “arrhythmic” presentation of peripartum cardiomyopathy: case series and critical review of the literature

**DOI:** 10.3389/fcvm.2024.1362692

**Published:** 2024-03-14

**Authors:** Giovanni Peretto, Emanuele Micaglio, Giuseppe Ciconte, Marianna Maia, Martina Luzzi, Marianna Cariello, Adele Gabriella Rosa Bonfanti, Davide Lazzeroni, Luigi Anastasia, Paolo Cavoretto, Alaide Chieffo, Paolo Della Bella, Carlo Pappone

**Affiliations:** ^1^ Cardiac Electrophysiology and Clinical Arrhythmology Department, IRCCS San Raffaele Scientific Institute, Milan, Italy; ^2^ Disease Unit for Myocarditis and Arrhythmogenic Cardiomyopathies, IRCCS San Raffaele Scientific Institute, Milan, Italy; ^3^ School of Medicine, Vita-Salute San Raffaele University, Milan, Italy; ^4^ Department of Cardiac Electrophysiology and Clinical Arrhythmology, IRCCS Policlinico San Donato, Milan, Italy; ^5^ Department of Cardiology, IRCCS Fondazione Don Carlo Gnocchi, Parma, Italy; ^6^ Department of Obstetrics and Gynecology, IRCCS San Raffaele Scientific Institute, Milan, Italy; ^7^ Interventional Cardiology Unit, IRCCS San Raffaele Scientific Institute, Milan, Italy

**Keywords:** PPCM, pregnancy, ventricular arrhythmias, genetic predisposition to adverse cardiac outcomes, multimodal diagnostic approach, multidisciplinary management

## Abstract

Peripartum Cardiomyopathy (PPCM) is a polymorphic myocardial disease occurring late during pregnancy or early after delivery. While reduced systolic function and heart failure (HF) symptoms have been widely described, there is still a lack of reports about the arrhythmic manifestations of the disease. Most importantly, a broad range of unidentified pre-existing conditions, which may be missed by general practitioners and gynecologists, must be considered in differential diagnosis. The issue is relevant since some arrhythmias are associated to sudden cardiac death occurring in young patients, and the overall risk does not cease during the early postpartum period. This is why multimodality diagnostic workup and multidisciplinary management are highly suggested for these patients. We reported a series of 16 patients diagnosed with PPCM following arrhythmic clinical presentation. Both inpatients and outpatients were identified retrospectively. We performed several tests to identify the arrhythmic phenomena, inflammation and fibrosis presence. Cardiomyopathies phenotypes were reclassified in compliance with the updated ESC guidelines recommendations. Arrhythmias were documented in all the patients during the first cardiological assessment. PVC were the most common recorder arrhythmias, followed by VF, NSVT, AF, CSD.

## Introduction

Peripartum cardiomyopathy (PPCM) is a rare myocardial disease occurring during late pregnancy or early postpartum period (1). Because of the frequent finding of reduced systolic dysfunction and heart failure (HF), PPCM is currently classified as a variant of dilated cardiomyopathy (DCM) (2). Consistently, the European Society of Cardiology (ESC) (3, 4) has adopted the revised version of the very earliest proposed diagnostic criteria (5, 6), namely: (1) development of HF from one month before delivery to the following five months—a narrow timeframe, which has been subsequently extended (1); (2) absence of an evident alternative cause other than pregnancy; (3) absence of known heart diseases diagnosed before the pregnancy; (4) LV ejection fraction (LVEF) < 45%, as defined by transthoracic echocardiogram.

While the DCM phenotype and associated mechanical manifestations are widely characterized in PPCM, to date the arrhythmic presentation of the disease is still under-investigated. In fact, although a broad range of tachy- and brady-arrhythmias have been described in patients with PPCM (1), most of the current knowledge relies on case reports and small-sized studies. The aim of the current review is: (1) to describe a series of patients evaluated for clinically-suspected PPCM following arrhythmic presentation; (2) to summarize the status of the art about the arrhythmic manifestations of PPCM.

## Case series

### Methods

We present a series of *n* = 16 consecutive patients evaluated for clinically-suspected PPCM at two centers specialized in arrhythmia management. Both inpatients and outpatients were identified retrospectively, based on the following screening criteria: 1) female sex in childbearing age (15 to 45 years); 2) first clinical presentation with arrhythmias (including bradyarrhythmias and either supraventricular or ventricular tachyarrhythmias), as documented either during pregnancy or in the 6 months after delivery; and: 3) lack of known cardiological history beforhead. In addition, in keeping with the local standard of care, multimodal diagnostic workup and multidisciplinary management were applied, respectively, to clarify the underlying diagnosis and enable patient-tailored treatment choices. In detail, on top of laboratory exams, transthoracic echocardiogram, 12-lead ECG and inhospital telemonitoring/outpatient Holter ECG monitoring, advanced diagnostic workup included one or more of the following exams: cardiac magnetic resonance (CMR) with late gadolinium enhancement (LGE) and additional sequences to investigate structural diseases (T2-weighted sequences, fat-sat sequences, parametric mapping whenever applicable); genetic test by next-generation sequencing to screen for cardiomyopathic gene variants (CGVs); histology exams, including hematoxylin-eosin and trichrome assays to detect myocardial inflammation and fibrosis, as well as immunohistochemistry analysis to further characterize the inflammatory infiltrates; 18-F fluorodeoxyglucose positron emission tomography (FDG-PET) scan, to screen for cardiac sarcoidosis in suspected cases; and electroanatomical map (EAM), to characterize the arrhythmogenic substrates in patients with clinical indication to catheter ablation. Cardiomyopathic phenotypes were reclassified in compliance with the updated (2023) ESC guideline recommendations (7).

But for the restrictions applied for pregnancy and lactation timeframes, all patients were offered optimal guideline-based medical therapy. Implantation of cardiac devices, as well as catheter ablation of arrhythmias, were in keeping with the current recommendations. At both centers, regular follow-up took place at dedicated outpatient settings for cardiomyopathy. The content of this report is fully compliant with the Declaration of Helsinki, and all patients signed informed consent to be enrolled in a research registry.

SPSS Version 20 (IBM Corp., Armonk, New York) was used for statistical analysis. Continuous variables were expressed as mean or median with standard deviation (SD) or range, depending on the distribution of data, as assessed by the Shapiro-Wilk's test. Categorical variables are reported as counts and percentages. Because of the small sample size and the absence of a prespecified study design, no statistical models were introduced for risk stratification, and no *p*-values were presented for comparison between groups.

### Results: clinical presentation

The series includes 16 women (mean age 31 years, range 24–36; 88% Caucasian), of whom 14 (88%) presented with symptoms, and were managed as inpatients. In detail, their clinical presentation was: cardiocirculatory arrest (*n* = 1), syncope (*n* = 1), palpitation (*n* = 5), dyspnea (*n* = 4), asthenia (*n* = 2), and chest pain (*n* = 1). The first clinical manifestation occurred during the third trimester of pregnancy in *n* = 3 cases (19%), and after delivery (median 4, range 1–6 months) in the remaining 13 (81%).

The key clinical features of the case series are show in Table 1. Obstetric history was unremarkable, except for two cases of twin pregnancy (13%, including one case occurring following *in-vitro* fertilization). No other patients had infertility or history of radiation exposure. The cardiovascular risk profile of the sample was generally low: in particular, there were no diabetic patients, and hypertension with criteria for preeclampsia was found in one single case (6%). Also, only two patients (13%) reported family history of sudden cardiac death (SCD) or cardiomyopathy.

**Table 1:** Key clinical features of the case series (*n* = 16).

ID	Age (year)	Obstetric history	Presentation	Rhythm /conduction disorders	Phenotype	LVEF (%)	Diagnostic workup	Final diagnosis	Cardiac device	Treatment	Outcomes
P01	35	Uncomplicated VD	Dyspnea	PVC, AF, RBBB	DCM	20	HE, GT	Undefined—class 3 variant in *VCL* gene	ICD (primary prevention)	Losartan	HTx
P02	33	Uncomplicated VD	Dyspnea	VT, incomplete RBBB, epsilon waves	DCM/ACM	25	GT	Genetic—class 5 variant in *TTN* gene	None (ICD refused)	None (refused)	SCD
P03	36	Uncomplicated CD	Dyspnea	NSVT, PVC	DCM	55	CMR, HE	Systemic sclerosis	None	Ramipril, bisoprolol, prednisone, MMF	Uneventful
P04	24	Uncomplicated VD	Chest pain	PVC	NDLVC	65	CMR, GT	Genetic—class 4 variant in *DSP* gene	ILR	Metoprolol	Uneventful
P05	30	Uncomplicated VD	Palpitation	NSVT, PVC	NDLVC	25	CMR, HE, EAM	Inflammatory (lymphocytic, virus-negative)	None	Losartan, propafenone, anakinra	PVC catheter ablation, LVEF recovery up to 58%
P06	26	Uncomplicated VD	Syncope	VT, NSVT, PVC, AF	DCM	30	CMR, EAM	Undefined (fibrotic)	ICD (secondary prevention)	ARNI, sotalol	AF catheter ablation, Mitraclip, LVEF recovery up to 48%
P07	33	Uncomplicated VD	Dyspnea	PVC, LBBB	DCM	32	GT	Undefined—class 3 variant in *FLNC* gene	CRT-D (primary prevention)	Bisoprolol	Uneventful
P08	24	Uncomplicated VD	Palpitation	PVC, WPW	Normal	60	CMR, EAM, GT	Arrhythmogenic mitral valve prolapse—class 3 variant in *LAMA4* gene	ICD (VF induced by PVS)	Fecainide	WPW catheter ablation; ablation of trigger PVC
P09	29	Twin pregnancy, UD	Asymptomatic	NSVT, PVC	NDLVC	59	CMR, HE, PET, EAM	Inflammatory (lymphocytic, virus-negative)	None	Sotalol, prednisone, azathioprine	PVC catheter ablation
P10	27	UD, premature	Palpitation, chest pain	PVC	NDLVC	59	CMR, HE	Inflammatory (lymphocytic, low-dose parvovirus B19)	ILR	Ramipril, bisoprolol, prednisone, azathioprine	Uneventful
P11	29	Twin pregnancy (ICSI), uncomplicated VD	Asymptomatic	NSVT, PVC	NDLVC	44	CMR, HE, EAM, GT	Undefined—class 3 variant in *DSP* gene	ICD (fast NSVT, extensive LGE, patient preference)	Metoprolol	ICD shock, VT catheter ablation, LVEF recovery up to 55%
P12	36	Uncomplicated VD	Palpitation	NSVT, PVC	NDLVC	60	CMR, EAM, GT	Inflammatory—class 3 variants in *DES*, *FLNC*, and *DMD* genes	ICD (fast NSVT, extensive LGE, genetic test)	Metoprolol, spironolactone, bromocriptin	PVC catheter ablation
P13	32	Uncomplicated CD	CCA	VF, PVC, WPW	Normal	50	EAM, GT	J-wave syndrome	Subcutaneous ICD (secondary preention)	Metoprolol, hydroquinidine	ICD shock for VF triggered by PVC; PVC and WPW catheter ablation
P14	36	Uncomplicated CD	Extreme asthenia	PVC, LBBB	DCM	40	CMR, PET, HE	Inflammatory (lymphocytic, virus-negative)	None	Bisoprolol	LVEF recovery up to 48%
P15	36	Uncomplicated CD	Asthenia	VT, PVC	NDLVC	65	CMR, EAM	Undefined (fibrotic, low-dose parvovirus B19)	ICD (secondary prevention)	Flecainide	Uneventful
P16	34	Uncomplicated VD	Palpitation	NSVT, PVC	NDLVC	57	CMR, HE, GT	Undefined (fibrotic)—class 3 variants in *DSP* and *RYR2* genes	ILR	Flecainide	Upgrade to ICD after fast NSVT causing syncope

Baseline clinical features, treatment and outcomes are shown for patients (*n* = 16) with the arrhythmic variant of PPCM.

ACM, arrhythmogenic cardiomyopathy; AF, atrial fibrillation; CCA, cardiocirculatory arrest; CD, Cesarean delivery; CMR, cardiac magnetic resonance; CRT-D, cardiac resynchronization therapy with defibrillator; DCM, dilated cardiomyopathy; DES, desmin; DMD, Duchenne's muscle dystrophy; DSP, desmoplakin; EAM, electroanatomical map; FLNC, filamin C; GT, genetic test; HE, histology exam; HTx, heart transplant; ICD, implantable cardioverter defibrillator; ICSI, intra cytoplasmatic sperm injection; ILR, implantable loop recorder; LAMA4, laminin subunit alptha-4; LBBB, left bundle branch block; LGE, late gadolinium enhancement; LVEF, left ventricular ejection fraction; NDLVC, nondilated left ventricular cardiomyopathy; NSVT, nonsustained ventricular tachycardia; PET, positron emission tomography; PPCM, peripartum cardiomyopathy; PVC, premature ventricular complexes; PVS, programmed ventricular stimulation; RBBB, right bundle branch block; RYR2, ryanodine receptor-2; SCD, sudden cardiac death; TTN, titin; VCL, vinculin; VD, vaginal delivery; VF, ventricular fibrillation; VT, ventricular tachycardia; WPW, Wolff-Parkinson-White.

Arrhythmias were documented in all patients at the time of first cardiological assessment after clinical presentation. In detail, premature ventricular complexes (PVC) were the most commonly recorded arrhythmia (median daily burden 1,128, range 322–21,960; short-coupled in two cases only), and showed dominant right bundle branch block morphology suggesting LV origin in 11/16; cases (69%). Other arrhythmias included ventricular fibrillation (VF) causing out-of-hospital cardiocirculatory arrest (*n* = 1), sustained ventricular tachycardia (VT; *n* = 2), nonsustained ventricular tachycardias (NSVT; *n* = 7), atrial fibrillation (AF; *n* = 1), Wolff-Parkinson-White syndrome (WPW; *n* = 2, incidental diagnosis), and conduction system disorders (CSD; *n* = 4). By the end of the baseline workup, most patients (88%) had more than one arrhythmia type documented.

### Results: diagnostic workup and clinical management

**Figure 1:**
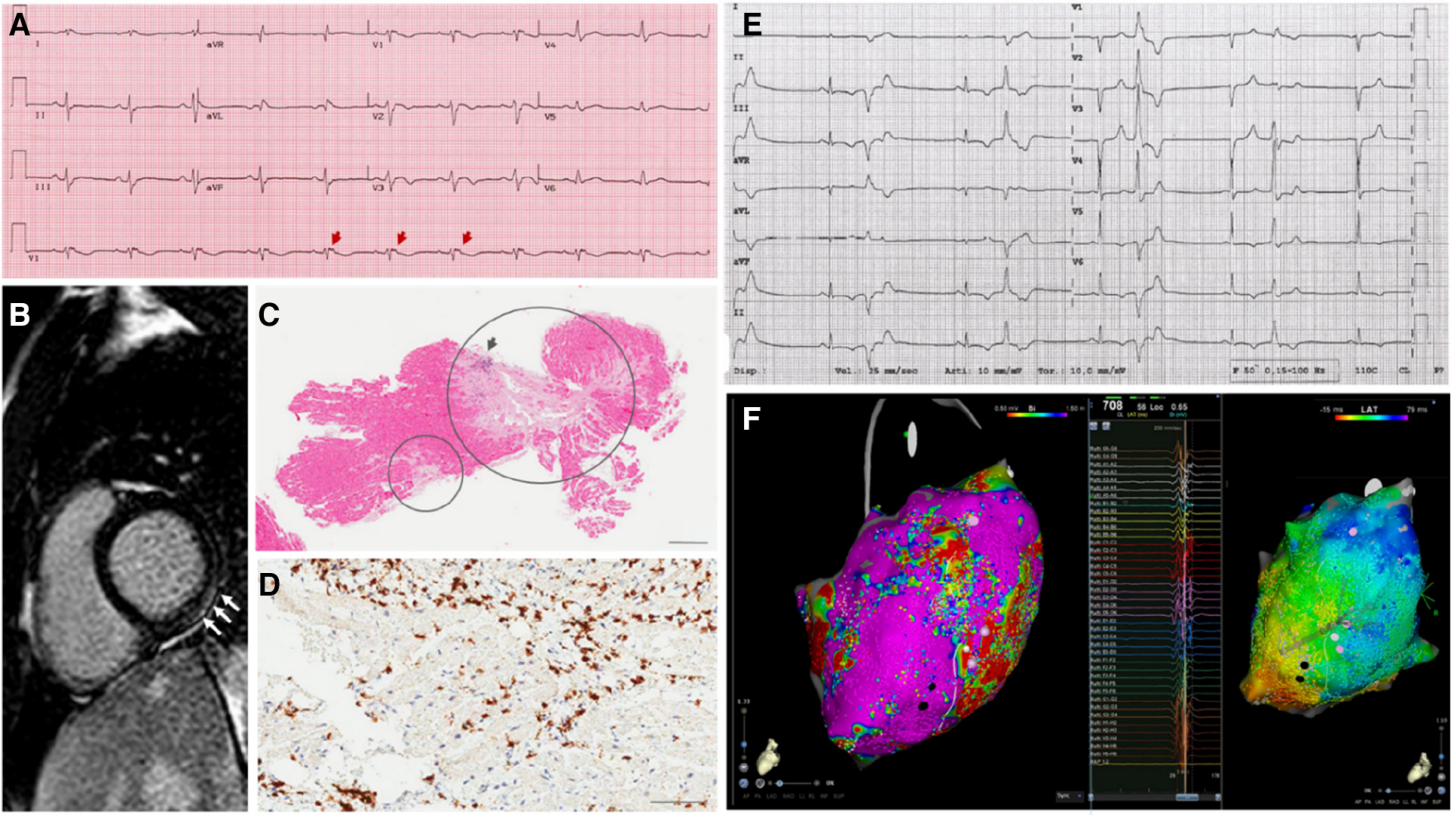
Representative examples of the diagnostic workup in the case series. The main results of diagnostic findings are shown. (**A**) 12-lead electrocardiogram in a patient (P02) with signs of arrhthmogenic cardiomyopathy, including negative T-waves in anterior precordial leads, and epsilon waves (arrows). (**B**) Strial of late gadolinium enhancement involving the basal segments of the inferolateral left ventricular wall (arrows), in a patient (P15) with nondilated phenotype. (**C**) Endomyocardial biopsy findings in a patient (03) with subsequent diagnosis of systemic sclerosis. Extensive areas of replacement fibrosis are shown (circles) on hematoxylin-eosin assay. (**D**) Immunohistochemical analysis on endomyocardial biopsy show >7/mm^2^ CD3-positive T-lymphocytes, meeting the diagnostic criteria for active-phase myocarditis in a patient (P09) with arrhythmic presentation. (**E**) 12-lead recording of polymorphic premature ventricular complexes in a patient (P04) with underlying mitral valve prolapse an nonischemic scar in the left ventricle. (**F**) High-density electroanatomical maps of the left ventricular epicardium (CARTO system, Biosense Webster; Octaray multielectrode catheter), including low-voltage areas (voltage map—on the left) and late potentials (activation map during sinus rhythm—on the right) involving the inferolateral wall, in a patient (P11) undergoing catheter ablation of a drug-refractory ventricular tachycardia.

At presentation, the mean LVEF was 47% (range 20%–65%), and phenotype was consistent with DCM in 6 patients (38%), non-dilated LV cardiomyopathy (NDLVC) in 8 (50%), and no criteria for structural disease in *n* = 2 (13%). Multimodality diagnostic workup included CMR (*n* = 13; 81%), genetic test (*n* = 9; 56%), histology (*n* = 8; 50%), FDG-PET scan (*n* = 2; 13%), and EAM (*n* = 8; 50%). Overall, a mean of 2.5 exams per patient on top of baseline echocardiogram were required to identify the final diagnosis, which was: defined genetic cardiomyopathy (*n* = 2), myocarditis (*n* = 5), systemic sclerosis (*n* = 1), arrhythmogenic mitral valve prolapse (*n* = 1), and J-wave syndrome (*n* = 1). Representative examples of the diagnostic workup are shown in Figure 1. The median time from clinical onset of final diagnosis was 18 (range 9–42) months, with no patients being diagnosed during pregnancy.

On top of standard medical treatment, including betablockers and renin-angiontensin-aldosterone-inhibitors in the postpartum period, antiarrhythmic agents were used in 6 patients (38%). Out of 7 women choosing breastfeeding, 4 had medical treatment temporarily interrupted during lactation. One patient (6%) received bromocriptine, and 4 (25%) underwent immunosuppressive therapy to target myocardial inflammation. Before discharge, implantable devices were placed in 11 patients (69%), including cardioverter defibrillators (ICD; *n* = 6, of whom 1 subcutaneous) and loop recorders in (ILR; *n* = 5). As per local standard practice, no patients received wearable cardioverter defibrillators (WCD). Because of uncontrolled psychiatric comorbidity, one patient refused any kind of therapy, including ICD implant.

### Results: outcomes

All patients had uncomplicated pregnancy, including *n* = 1 preterm (6%) and *n* = 4 caesarean deliveries (25%). No health issues were reported in children. By a median follow-up of 7 (range 2–34) years, *n* = 4 patients (25%) experienced major adverse outcomes including SCD from cardiocirculatory arrest (*n* = 1), appropriate ICD shocks (*n* = 2), and end-stage heart failure requiring heart transplantation (*n* = 1). In addition, 6 patients (38%) requires catheter ablation of arrhythmias (PVC, *n* = 4; VT, *n* = 1; AF, *n* = 1, WPW, *n* = 2). Follow-up was uneventful in the remaining patients, and was remarkable for left ventricular reverse remodelling (LVRR) with improvement in LVEF in four of the six cases with DCM (67%). Three patients (19%) subsequently underwent new uncomplicated pregnancy and delivery, without LVEF decrease. Four patients underwent exercise stress test late after delivery without complications.

The relationships between baseline features and outcomes are summarized in Table 2. The variales showing better association with the occurrence of major adverse outcomes included clinical presentation with sustained VT or VF, and presence of notable ECG abnormalities as epsilon- and J-waves (incidence of major adverse outcomes: 2/2 vs. 2/14, *p* = 0.05, for both variables). To be noted that the adverse outcomes (VF for P08, ICD shock for P13, see Table 1) were not related to WPW, which was previously treated via catheter ablation. A weaker association (*p* < 0.30) was found with LVEF < 50%, CSD, supraventricular arrhythmias, and abnormal *T*-waves (Table 2). For other clinically-relevant variables, such as genotypes and LGE, any reliable association analyses were prevented by the very small sample size. It should be highlighted that no adverse events occurred in patients receiving either immunomodulatory or prolactin-inhibitory therapy (0/5 vs. 4/11, *p* = 0.24).

**Table 2:** Relationships between clinical features and outcomes.

Feature	Prevalence, *n* (%)	Major adverse outcomes^a^	Need for ICD or ablation	LVRR
Age > 30 years	9 (56)	3/9 vs. 1/7	6/9 vs. 6/7	1/9 vs. 3/7
African ethnicity	2 (13)	0/2 vs. 4/14	1/2 vs. 11/14	1/2 vs. 3/14
Family history of SCD/CMP	2 (13)	1/2 vs. 3/14	2/2 vs. 10/14	0/2 vs. 4/14
CVRF	6 (38)	1/6 vs. 3/10	5/6 vs. 7/10	2/6 vs. 2/10
BMI > 25 kg/m^2^	5 (31)	1/5 vs. 3/11	3/5 vs. 9/11	1/5 vs. 3/11
Twin pregnancy	2 (13)	1/2 vs. 3/14	2/2 vs. 10/14	1/2 vs. 3/14
Extracardiac comorbidity	5 (31)	2/5 vs.2/11	4/5 vs. 8/11	1/5 vs. 3/11
Syncope	2 (13)	1/2 vs. 3/14	2/2 vs. 10/14	1/2 vs. 3/14
Sustained VT or VF	2 (13)	2/2 vs. 2/14	2/2 vs. 10/14	0/2 vs. 4/14
NSVT	7 (44)	1/7 vs. 3/9	6/7 vs. 6/9	3/7 vs. 1/9
PVC > 1,000/24 h	10 (63)	2/10 vs. 2/6	7/10 vs. 5/6	2/10 vs. 2/6
Left ventricular PVC	13 (81)	3/13 vs. 1/3	9/13 vs. 3/3	3/13 vs. 1/3
Supraventricular arrhythmias	4 (25)	2/4 vs. 2/12	4/4 vs. 8/12	1/4 vs. 3/12
CSD	4 (25)	2/4 vs. 2/12	3/4 vs. 9/12	1/4 vs. 3/12
Epsilon/*J*-waves	2 (13)	2/2 vs. 2/14	2/2 vs. 10/14	0/2 vs. 4/14
*T*-wave abnormalities	10 (63)	4/10 vs. 0/6	9/10 vs. 3/6	3/10 vs. 1/6
High natriuretic peptides	4 (25)	1/4 vs. 3/12	2/4 vs. 10/12	1/4 vs. 3/12
DCM	6 (38)	2/6 vs. 2/10	4/6 vs. 8/10	2/6 vs. 2/10
NDLVC	8 (50)	1/8 vs. 3/8	6/8 vs. 6/8	2/8 vs. 2/8
LVEF < 50%	7 (44)	3/7 vs. 1/9	6/7 vs. 6/9	4/7 vs. 0/9
Mitral valve prolapse	2 (13)	0/2 vs. 4/14	2/2 vs. 10/14	1/2 vs. 3/14
LGE on CMR	10/12 (83)	1/10 vs. 0/2	6/10 vs. 2/2	3/10 vs. 1/2
Myocardial inflammation	7/12 (58)	1/7 vs. 0/5	4/7 vs. 4/5	3/7 vs. 1/5
Class 4/5 gene variants Class 3/4/5 gene variants	2/9 (22) 8/9 (89)	1/1 vs. 3/7 3/8 vs. 1/1	1/1 vs. 7/7 7/8 vs. 1/1	0/1 vs. 1/7 1/8 vs. 0/1
Positive PVS	2/4 (50)	0/2 vs. 1/2	2/2 vs. 2/2	0/2 vs. 1/2
RAAS-inhibitors	5 (31)	1/5 vs. 3/11	3/5 vs. 9/11	2/5 vs. 2/11
Betablockers	10 (63)	2/10 vs. 2/6	6/10 vs. 6/6	3/10 vs. 1/6
Antiarrhythmics	7 (44)	1/7 vs. 3/9	7/7 vs. 5/9	2/7 vs. 2/9
PD-treatment	5 (31)	0/5 vs. 4/11	3/5 vs. 9/11	1/5 vs. 3/11

Relationships between baseline clinical features and outcomes are shown for PPCM patients.

BMI, body mass index; CMP, cardiomyopathy; CMR, cardiac magnetic resonance; CSD, conduction system disorders; CVRF, cardiovascular risk factors; DCM, dilated cardiomyopathy; ICD, implantable cardioverter defibrillator; LGE, late gadolinium enhancement; LVEF, left ventricular ejection fraction; LVRR, left ventricular reverse remodelling; NDLVC, nondilated left ventricular cardiomyopathy; NSVT, nonsustained ventricular tachycardia; PD, pathophysiology-driven; PPCM, peripartum cardiomyopathy; PVC, programmed ventricular complexes; PVS, programmed ventricular stimulation; RAAS, renin-angiotensin-aldosterone system; SCD, sudden cardiac death; VF, ventricular fibrillation; VT, ventricular tachycardia.

^a^ Major adverse outcomes include cardiac death, hear transplant, or malignant ventricular arrhythmias (sustained VT/VF or appropriate ICD therapy).

## Critical review of the literature

### Epidemiology

The global incidence of PPCM is 1 in 1,000 worldwide, with peak values in northern Nigeria (1:100) and Haiti (1:300) (8). Recognized risk factors for PPCM include African American ethnicity, maternal age over 30 years, chronic hypertension, pregnancy-associated-hypertensive conditions as preeclampsia, anemia, and prolonged use of beta-agonist tocolytics during threatened preterm labor (2, 8, 9).

Our report was notable for including women with no prior cardiological history, the majority of whom being Caucasian (88%). In addition, we hereby provided extensive characterization of patients with arrhythmias recorded during baseline workup, either at clinical presentation or immediately after. Remarkably, DCM phenotype accounted for <50% of our cohort, so that an “arrhythmic variant” of PPCM was hereby described. In the largest study on a population of 9,841 patients with classically-defined PPCM, the overall prevalence of arrhythmias was 19% (10). Among them, ventricular arrhythmias were the most common ones (4% for VT, 1% for VF), followed by supraventricular arrhythmias (1.3% for AF, 0.5% each for atrial flutter and atrial tachycardia, 0.3% for paroxysmal reentry tachycardia including WPW) and 2.5% of CSD mainly including left bundle branch blocks (10, 11). No conflicting data emerged from our series, except for PVC, which was the most common arrhythmia in our experience (15/16) in contrast with the 0.1% prevalence reported so far for both atrial and ventricular ectopies (10, 11). In this setting, we attempt to bridge a knowledge gap (10, 11), by providing data about daily burden (widely variable in range 322–21,960), and morphology (mainly right bundle branch block, suggesting LV origin, as expected in PPCM).

### Pathophysiology

The pathophysiology of arrhythmias in PPCM reflects the multifactorial nature of the disease, whose dominant mechanisms are summarized in Table 3. Briefly, hemodynamic changes, autonomic dysregulation, electrolyte imbalances, systemic inflammation, metabolic and hormonal effects have been described, either as a substrate or triggering events for HF and arrhythmias related to PPCM (2, 14, 15).

**Table 3:** Pathophysiological mechanisms of PPCM.

Mechanism class	Mechanism type	Effects on PPCM	Effects on heart rhythm	References
Hemodynamic changes	↑ blood volume (+30%)	↑ LVEDP, ↑ LVEDV	Sinus tachycardia, arrhythmias from volume/pressure overload	(8, 11, 12)
	↑ stroke volume (+25%)	↑ LVEDP		
	↑ vascular peripheral resistances	↑ LEDVP, ↑ LVH		
Autonomic dysregulation	↑ adrenergic tone	↓ CFR, ↑HR, ↑Heart work, ↑HF, ↑LVEDP.	Sinus tachycardia, ectopic beats, adrenergic VA, enhanced reentry if preexisting accessory pathways or dual atrioventricular node physiology	(2, 11, 13)
Electrolyte imbalance	Hypokalemia	↑HR, ↑Heart work	Long QT, polymorphic VA	(13, 14)
Vascular abnormalities	Enhanced angiogenesis from ↑ serum PlGF, ↑ serum sFLt-1	Preeclampsia, ↑ LVEDP	Unknown	(2, 8, 15)
	Endothelial dysfunction	↓ Tissue repair	Unknown	
	Coronary microvascular dysfunction	Ischemia	Arrhythmias from myocardial ischemia and scarring	
Inflammation and immune dysregulation	↑ circulating proinflammatory cytokines (CRP, TNF-alpha, IL-6)	↓ dp/dt, ↑HF, ↑LVED P.	VA and bradyarrhythmias	(8, 11, 13, 16)
	Myocardial inflammation	DCM ↑Fibrosis, ↑HF, ↓Cardiac output	Hot-phase, inflammation-dependent arrhythmias; Cold-phase, scar-related arrhythmias	
	Viral genomes (i.e. EBV, CMV, HHV6, PVB19) within cardiac myocytes	Chronic DCM and heart failure (+1/3), ↑ cardiac interstitial inflammatory process	↑Vasodilatation, ↑LVP	
Hormonal effects	↑ prolactin secretion: reduction in STAT3 leads to prolactin cleavage in an antiangiogenic and proapoptotic isoform	↓Angiogenesis,↑Vasoconstriction, ↑ Systemic resistances which lead to ↑HF.	Arrhythmias (from the most common to the less): AF, VT, VF	(9, 11, 13)
	↑ levels of estradiol and progesterone	↑Vasodilatation, ↑LVEDP	Arrhythmias	
Metabolic dysregulation	Increased MHR and LDL	LV adverse remodeling due to ↑ oxidative stress	Arrhythmias	(2, 8, 11, 13)
	Increased adipogenesis	LV adverse remodeling due to ↑ oxidative stress	T-wave inversion, PVC, VT	
	Nutritional deficiencies	↓ dp/dt, LV adverse remodeling	Unknown	
Genetic background	Pathogenic or likely-pathogenic variants in cardiomyopathy-associated genes (*TTN*, *DSP*, *MYH6*, *MYH7*, *TPM1*, *VCL*, *RBM20*)	DCM, Myocardial inflammation	Ventricular arrhythmias: VT, VF	(16, 17)

The main pathophysiological mechanisms of PPCM are shown, including the relationships with arrhythmogenesis.

AF, atrial fibrillation; CFR, coronary flow reserve; CMV, cytomegalovirus; CRP, C-reactive protein; DCM, dilated cardiomyopathy; DSP, desmoplakin; dp/dt, contractility; EBV, Epstein-Barr virus; HF, heart failure; HR, heart rate; HV6, human herper virus-6; IL-6, interleukin-6; LDL, low-density lipoprotein; LV, left ventricle; LVEDP, left ventricular end-diastolic pressure; LVH, left ventricular hypertrophy; MHR, monocyte to high-density lipoprotein ratio; MYH6, myosin heavy chain-6; MYH7, myosin heavy chain-7; PlGF, placental growth factor; PPCM, peripartum cardiomyopathy; PVB19, parvovirus B19; PVC, premature ventricular complexes; RBM20, RNA binding motif-20; sFLt-1, soluble fms-like tyrosine kinase-1; STAT3, single transducer and activator of transcription-3; TNF-alpha, tumor necrosis factor-alpha; TPM1, tropomyosin-1; TTN, titin; VA, ventricular arrhythmias; VCL, vinculin; VF, ventricular fibrillation; VT, ventricular tachycardia.

As an alternative to the multisystemic dysregulation hypothesis, it has been suggested that latent preexisting myocardial diseases, including but not limited to myocarditis and primary cardiomyopathies, may retain a primary role in the disease pathophysiology (2, 12). In this setting, the current definition of PPCM (2, 3) is challenging, since a preexisting undiagnosed disease may be simply unmasked during pregnancy or after delivery. In a study (9), almost one third of PPCM patients showed biopsy-proven cardiotropic viral genomes, suggesting that DCM and HF may occur as late manifestations of chronic myocarditis. An increased risk of preeclampsia has been reported also in association with COVID-19 infection (18). Autoimmune virus-negative myocarditis has also been described as a driver mechanism in PPCM (2), also because of the microchimerism from fetus-derived cells during the immune-suppressed pregnant state (19). As known, myocarditis may account for a broad range of arrhythmias, complicating both the inflammatory and the postinflammatory phases of the disease, even in the patients with preserved LVEF (20, 21). In our series, myocarditis was detected either by CMR or EMB in 7 of 16 patients (44%). As the only viral genome found in the myocardium, parvovirus B19 (load < 500 copies/mcg) was infrequently found (Table 1). While prior studies failed in demonstrating higher rates of EMB-proven myocarditis among PPCM cases (22, 23), the role of myocardial inflammation as an arrhythmogenic substrate is still to be investigated.

In turn, the genetic basis of PPCM has been recently revealed (16, 24). Historically, PPCM has been differentiated from primary DCM because of its idiopathic, non-familial, non-genetic substrate (3, 4, 9). For instance, distinct cellular pathways downstream prolactin have been described as cardiomyopathic susbstrate specific of PPCM (25). However, in a recent study on 172 women with PPCM (17), truncating variants in genes predisposing to DCM were identified in 26 cases (15%). In this setting, volume overload and other systemic changes associated with pregnancy, may act as accelerating factors in sensitive genotypes. The main reports involved genes encoding titin (*TTN*), desmoplakin (*DSP*), alpha myosin heavy chain protein (*MYH6*), tropomyosin (*TPM1*), vinculin (*VCL*) and lamin A/C (*LMNA*), which constitute key structural and functional components for the cytoskeleton organization (7, 17, 26). Consistently, we detected CGVs in 8 of the 9 gentoyped patients (89%). While two patients only (13%) carried CGVs with a compelling pathogenic role (class 4/5), the hemodynamic changes associated with physiological pregnancy may have unmasked a concealed cardiomyopathic substrate even in the remaining subjects. Similar effects have been described for women carrying *TTN* truncating variants, where pregnancy has been described as a “second hit” for the classic PPCM presentation (17). In this setting, the presence and type of arrhythmias may strongly depend on the genotype. For instance, cytoskeletal genes may predispose to maladaptive evolution towards DCM, whereas desmosomal genes towards ventricular arrhythmias and myocardial inflammation (17, 27). In turn, mutations in the *LMNA* gene may account for both brady- and tachyarrhythmias, much earlier than overt LV systolic dysfunction occurs (28). Preliminary evidence suggests that life-threatening arrhythmias in the peripartum may be associated even with Brugada syndrome or long QT syndrome (29–31). Dedicated studies are needed to add confirmatory evidence in this setting.

### Multimodality diagnostic workup

In compliance with the current standards, PPCM should be suspected every time signs or symptoms of cardiac disease are found for the first time in a pregnant woman (2, 3). In the “classic” DCM phenotype, PPCM may be easily detected by routine transthoracic echocardiogram (8). In particular, a number of parameters may differentiate pregnancy-associated physiological findings from maladaptive PPCM changes by ultrasounds (32–35). However, diagnosis may be more challenging following clinical onset of arrhythmias: as noted above, many of the patients included in our report (63%) had either NDLVC or normal phenotype. While the finding of LVEF < 45% was uncommon, and the diagnosis of the classic variant of PPCM was subsequently not met, all patients in our series had documented arrhythmias with or without signs of associated muscle disease (Table 1). To be noted, only two patients in our series (13%) had relatives known for SCD or cardiomyopathy, in line with the 15% prevalence reported in a published German registry (36). While a number of arrhythmogenic conditions, such as WPW, were likely preexisting and simply unmasked by pregnancy, diagnostic assessment was challenging for most women. As a uniform tract, arrhythmias were documented, and diagnosed for the first time either during pregnancy or by 6 months after delivery, so that an “arrhythmic” variant of PPCM is hereby proposed. Importantly, arrhythmic manifestations occurred irrespectively of LVEF values (Table 1). In this context, the overlap with arrhythmogenic cardiomyopathy, channelopathies and inflammatory heart diseases is more demanding as compared with DCM. As currently suggested for many arrhythmogenic cardiomyopathies (7, 37), even in our experience a multimodality diagnostic approach was useful in characterizing the disease. Table 4 summarizes the spectrum of diagnostic techniques available to detect cardiomyopathic substrates in PPCM.

**Table 4:** Diagnostic workup and findings in pregnancy and PPCM.

Exam	Pregnancy-associated findings	PPCM-associated findings	Caveats in pregnancy	References
ECG and Holter ECG	Sinus tachycardia (30–40%) Leftward shift of the QRS axis	*T*-wave inversion (70%) Long QT (44%) Brugada pattern (reports) ST-segment abnormalities (14%) AV blocks (reports) LBBB (1%) Atrial fibrillation (reports) Other paroxysmal supraventricular arrhythmias (reports) Ventricular arrhythmias (PVC, NSVT,VT)	None	(10, 11, 14, 34)
Echocardiogram	Increased cardiac chambers volume. LV hypertrabeculation. Preserved systolic function	Systolic dysfunction, with LVEF < 45% LVEDD > 60mm–64mm LVFS < 16% LAVi > 30 ml/m^2^ LVGLS > 11%, LVGCS > 10% RVFAC < 31% Mitral regurgitation	None	(32–35)
Blood exams	Normal natriuretic peptides and troponin	Natriuretic peptide elevation (NTproBNP > 300 pg/ml, BNP > 100 pg/ml), Troponin elevation (suggests myocarditis, spasm or SCAD)	None	(4, 9, 33, 38, 39)
CMR	Increased LVEDV, RV size, LAVi. LV hypertrabeculation. Unchanged LVEF, RVEF. Absence of LGE and cardiomyopathy-associated tissue abnormalities	LGE →DCM findings (presence of midwall septal stria) Mural thrombi T2-weighted abnormalities	Avoid IV gadolinium administration if not necessary (however, both the diagnostic and prognostic values of the exam may be limited). Discontinue lactation for 24h after IV gadolinium administration	(3, 7, 40–43)
Coronary angiography, CT scan	Normal epicardial coronary arteries	ACS, spasm or SCAD	Radiation exposure, contrast toxicity, procedural risk	(7, 13, 14)
EMB	Normal cardiac myocytes. Absence of fibrosis, inflammation, storage diseases. Microvascular remodelling	Myocardial inflammation, LV/RV hypertrophy. Replacement, interstitial, perivascular fibrosis	Radiation exposure, procedural risk	(9, 22, 23)
FDG-PET	Normal FDG uptake	Possible sarcoidosis pattern	Radiation exposure	(7, 44, 45)
Electroanatomical mapping	Normal endocardial voltage. Absence of late potentials	Low-voltage areas Late potentials suggesting underlying cardiomyopathy	Radiation exposure, contrast toxicity, procedural risk. Indication limited to patients with indication to catheter ablation of arrhythmias	(7, 46, 47)
Genetic testing		DCM shared background: pathogenic or likely-pathogenic variants, mainly in *TTN*, *DSP*, *TPM1*, *MYH6*, *VCL*, and *LMNA* genes	Counselling for risk and family screening	(3, 7, 17, 48)

The main diagnostic findings expected in the arrhythmic variant of PPCM are shown.

ACS, acute coronary syndrome; AV, atrioventricular; BNP, brain natriuretic peptide; CMR, cardiac magnetic resonance; CT, computed tomography; DCM, dilated cardiomyopathy; DSP, desmoplakin; ECG, electrocardiogram; EMB, endomyocardial biopsy; FDG, fluprodeoxyglucose; IV, intravenous; LAV(i)=left atrial volume (indexed); LBBB, left bundle branch block; LGE, late gadolinium enhancement; LMNA, lamin A/C; LV, left ventricular; LVEDD, left ventricular end-diastolic diameter; LVEF, left ventricular ejection fraction; LVFS, left ventricular fractional shortening; LVGCS, left ventricular global circumferential strain; LVGLS, left ventricular global longitudinal strain; NSVT, nonsustained ventricular tachycardia; NTproNP, N-terminal brain natriuretic pepetide; PET, positron emission tomography; PPCM, peripartum cardiomyopathy; PVC, premature ventricular complexes; RV, right ventricular; RVFAC, right ventricular fractional area change; RVEF, right ventricular ejection fraction; SCAD, spontaneous coronary artery dissection; TPM-1, tropomyosin-1; TTN, titin; VCL, vinculin; VT, ventricular tachycardia.

In classic PPCM, sinus rhythm ECG may reveal signs suggestive for PPCM, like *T*-wave inversion in up to 70% of patients (14, 49). Cardiac biomarkers, such as natriuretic peptides BNP and NT-proBNP, are frequently elevated (9, 38, 39). Beyond hypokynesis, echocardiogram may show extensive remodeling of cardiac chambers and diastolic dysfunction (3, 34, 50, 51). Not infrequently, LV hypertrabeculation exceeding the degree expected during pregnancy is observed (8, 52). While functional mitral valve regurgitation in classic PPCM may occur secondarily to LV dilation, thickened leaflets and specific signs should call for mitral valve prolapse as an alternative source of arrhythmias (53).

Among second-level imaging techniques, CMR is currently considered as the gold standard in cardiomyopathies (7), and it is proven safe in pregnancy (3). Although no specific diagnostic criteria for PPCM have been described at CMR, most patients with classic PPCM phenotype had no evidence of LGE (40, 41). As opposed, we documented nonischemic LGE in almost all women with the arrhythmic variant of PPCM (Table 2). In this setting, distinct patterns of LGE may also point to specific diagnoses, such as primary DCM in the presence of midwall septal stria (7), myocarditis in association with subepicardial involvement of the inferolateral wall (42), and distinct variants of NDLVC in the presence of a ring-like appearance (7, 43). In addition, abnormalities on T2-weighted sequences enforce the suspicion of myocardial inflammation (54), which frequently deserves confirmation and further etiological characterization by EMB, as recommended in patients with myocarditis (42). Histology may also reveal tissue remodeling, fibrosis, and associated viral genomes (9, 22, 23). As an alternative to histology, FDG-PET may be particularly useful whenever cardiac sarcoidosis is clinically suspected, or implantable device-related artifacts prevent the interpretation of CMR (7, 44–55). Finally, in patients with clinical indication to catheter ablation, EAM may help identifying low-voltage areas or electrogram abnormalities suggestive for a cardiomyopathic substrate (46, 47). Whenever familial disease is suspected, or upstream workup suggests signs of a genetic disease, wide-spectrum genetic test should be strongly considered (3, 7, 48). Even in the absence of macroscopic substrate abnormalities, long QT syndrome, Brugada syndrome, catecholamine-related VT syndromes may account for concealed arrhythmogenic substrates (56, 57). In such heterogeneous scenarios, genotyping may help in reaching a definite diagnosis. In partial agreement with the published literature (16, 17), 50% of patients in our report had CGVs detected by genetic test. Nonetheless, because of the frequent finding of variants of unknown significance, diagnosing a genetically-proven cardiomyopathy was challenging in the majority of cases.

It is worth noting that, in our series, the average number of the above-mentioned second level exams was 2.5 per patient, thus allowing to reach the diagnosis by a median follow-up of 18 months from clinical onset, i.e., late after delivery. Our data indicate that diagnostic characterization for clinically-suspected PPCM may be complex and lengthy, and relies on multimodality workup.

**Figure 2:**
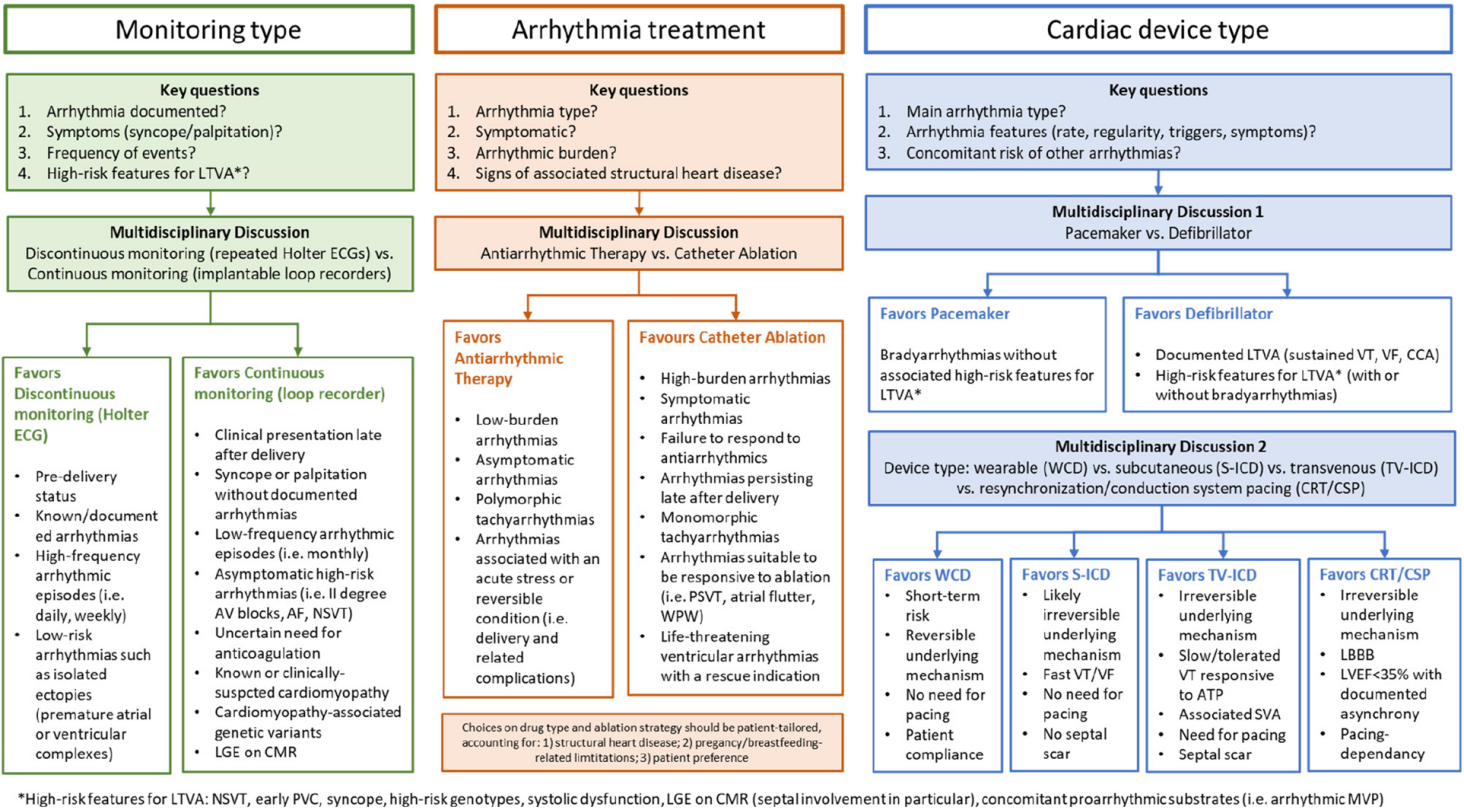
Clinical scenarios in the arrhythmic variant of PPCM. The main clinical challenges for multidisciplinary heathcare teams to manage patients with clinically-suspected peripartum cardiomyopathy and either proven or suspected arrhythmias are shown. AF, atrial fibrillation; ATP, anti-tachycardia pacing; AV, atrioventricular; CCA, cardiocirculatory arrest; CMR, cardiac magnetic resonance; CRT/CSP, cariac resynchronization therapy/conduction systema pacing; ECG, electrocardiogram; ICD, implantable cardioverter defibrillator (S, suubcutaneous; TV, transvenous); LBBB, left bundle branch block; LGE, late gadolinium enhancement; LTVA, life-threatening ventricular arrhythmias; LVEF, left ventricular ejection fraction; MVP, mitral valve prolapse, NSVT, nonsustained ventricular tachycardia; PSVT, paroxysmal supraventricular tachycardia; SVA, supraventricular arrhythmias; VF, ventricular fibrillation; VT, ventricular tachycardia; WCD, wearable cardioverter defibrillator; WPW, Wolff-Parkinson-White syndrome.

One last critical point concerns the detection of arrhythmic episodes. Since arrhythmias may arise suddenly, discontinuous monitoring by means of repeated Holter ECG of 24 or 48h registration, may result in significant underdetection of rhythm disorders (58). In our series, most patients had arrhythmias detected because of inhospital setting and continuous telemonitoring. For instance, NSVT episodes were detected in up to 7 of 16 patients (44%), in contrast to the 21% detected by Holter ECG in a published series (58). Given that continuous electrical monitoring techniques had demonstrated superiority to discontinuous monitoring in similar clinical scenarios (59), ILR may find application in selected cases considered at lower risk of SCD and no indication to ICD (60). In our series, one patient carrying ILR subsequently underwent upgrade to ICD because of fast NSVT episodes missed by Holter ECG. The proposed diagnostic algorithm for arrhythmia detection in PPCM is shown in Figure 2.

### Risk stratification

PPCM has an increasing incidence (8), and has been reported as the leading cause of maternal cardiovascular death (61), with mortality rates ranging from 1.3% inhospital, to 16% at 7 years (62, 63). Overall, VA are the most threatening manifestations of classic PPCM, accounting for up to 1 out of 4 cases of SCDs (13). Mortality of PPCM-patients experiencing arrhythmia is 2.1%, three-fold higher than without arrhythmias (11).

Instead, bradyarrhythmias are reported benign and self-limited in most cases (10). In fact, unless accompanied by ventricular arrhythmias, underlying diseases with adverse prognostic significance such as sarcoidosis and *LMNA* cardiomyopathy are unlikely (60).

As for the mechanical manifestations of the disease, LV reverse remodeling and full recovery of LVEF have been described in many patients during the postpartum period, with LVEF normalization rates of up to 71% by 6 months after delivery (64). Also in our series, LVEF at presentation was associated with higher recovery rates (Table 2), confirming the published data (8). In classic PPCM, additional prognostic factors for heart failure include LVEF below 45%, increased LV end-diastolic diameter, reduced LV strain parameters, right ventricular or biventricular dysfunction, and increased left atrial volume (32, 33, 65, 66). Also, women whose LV ejection fraction failed to return within the normal range after their first episode of PPCM showed an increased risk of PPCM recurrence in a subsequent pregnancy (67, 68). In this setting, NT-proBNP values ≥900 pg/ml were found as negative predictors of LV reverse remodeling (38), whereas a BNP value <100 pg/ml was found accurate in ruling out adverse events related to PPCM (39).

Even in the absence of an overt DCM phenotype, the identification of LGE on CMR, especially with a septal distribution pattern, can be predictive of both for SCD and end-stage heart failure (60, 69, 70). Advanced myocardial imaging may also identify mitral annular disjunction and additional prognostic signs for arrhythmogenic mitral valve prolapse (71). In this setting, the genetic test has a major impact on patient prognosis: in fact, in compliance with the updated guideline recommendations (7), the identification of “high risk” genotypes may significantly contribute to both the arrhythmic risk stratification and the clinician's decision of ICD implant. In our experience, while both medical treatment and ICD were refused by the patient, the only case of SCD occurred in a patient with overt DCM who harbored a pathogenic TTN truncating mutation (as reported in Table 1).

Remarkably, prognostic evaluation by disease-specific risk factors cannot be applied in the absence of a specific etiology (7, 60). As far as no comprehensive risk score calculators become available for PPCM from large multicenter studies, a multimodal and patient-tailored arrhythmic risk stratification strategy is strongly advised.

### Personalized treatment strategies

An evidence-based overview of the available treatment options to manage arrhythmias in PPCM is presented in Table 5. The traditional RAAS-inhibitors, as well as angiotensin receptor-neprilysin inhibitors and mineralocorticoid receptor antagonists, are contraindicated during pregnancy and can be only used in the post-partum period (2–4, 73), as occurred in our cases. Episodes of acute HF are managed by oxygen administration, fluid restriction, loop diuretics, nitrates and vasodilators as hydralazine (8, 9, 72). In severe cases, inotropes and mechanical circulatory support are needed (2, 8). Anticoagulants can be administered according to the current recommendations in patients with LVEF < 30%–35% (76, 77), in particular in the presence of risk factors for thromboembolic events, as in AF or LV hypertrabeculation (52).

**Table 5:** Treatment options for PPCM and related arrhythmias.

Therapy	Indication	First choice	Caveats in pregnancy/breastfeeding	References
Heart failure—DCM treatment				
ACE-inhibitors, ARB, ARNI, MRA	LV reverse remodeling	None (patient-tailored)	Contraindicated during pregnancy. Can be used only in postpartum	(3, 4)
Diuretics	Heart failure symptoms	Loop Diuretics	Avoid if hypertension/preeclampsia, for risk of reduced blood flow in the placenta	(8, 9, 72)
Vasodilators	Hypertension, acute heart failure	Hydralazine and nitrates	Adverse effect: SLE-like syndrome, fetal tachyarrhythmias	(8, 9, 72)
Inotropes	Acute heart failure	Dopamine and levosimendan	Only when the foreseen beneficial effects overweight risks	(72)
MCS	Acute heart failure refractory to inotropes	None (patient-tailored)	Only when the foreseen beneficial effects overweight risks	(2, 8, 72)
Arrhythmia management				
Betablockers	Supraventricular tachyarrhythmias, ventricular arrhythmias, LQTS and other arrhythmogenic diseases	metoprolol, sotalol, propranolol	Avoid atenolol, bisoprolol	(60, 73)
Calcium channel antagonists	Supraventricular tachyarrhythmias	verapamil	Favor non-dihydropiridinic agents	(59, 73)
Other antiarrhythmic drugs	Supraventricular tachyarrhythmias, ventricular arrhythmias	flecainide, digoxin	Avoid amiodarone and dronedarone (fetal hypothyroidism). Only when the foreseen beneficial effects overweight risks (teratogenic risk during the first trimester, abnormal growth development later)	(73–75)
Anticoagulants	AF, LVNC, of intracardiac thrombi/systemic embolism	LMWH, VKA, UFH	Avoid vitamin K antagonist in the first trimester (embryopathy). Prefer LMWH in the first trimester	(52, 76, 77)
Electrical cardioversion	Supraventricular tachyarrhythmias, ventricular arrhythmias	None	Only for hemodynamic unstable tachyarrhythmias	(60, 73)
Pacemakers	Irreversible symptomatic bradycardia due to third-degree or second-degree Mobitz type II heart block or severe sinus node dysfunction, with syncope or presyncope	None (patient-tailored)	Safer when implanted with fetus beyond 8 weeks gestation. Rule-out high-risk features for ventricular arrhythmias. Favor near-zero fluoroscopy procedures.	(73, 78–80)
Defibrillators	LVEF < 35% without reversibility features (primary prevention). Sustained VT episodes during pregnancy (secondary prevention)	None (patient-tailored)	Give no contraindications for future pregnancies. Prefer WCD with a bridge-to-recovery or bridge-to-decision indication. Avoid S-ICD if need for pacing is foreseen.	(3, 4, 81, 82)
Catheter ablation	Drug-refractory and/or poorly tolerated tachycardias.	None	If possible defer to the 2nd trimester or after delivery due to radiation exposure. Favor near-zero fluoroscopy procedures.	(46, 47, 73)
Pathophysiology-guided treatment				
Bromocriptine	Reduced LVEF heart failure	None	To be considered in combination with heart failure therapy and anticoagulation therapy	(83, 84)
Immunosuppressive therapy	Fulminant myocarditis. Chronic virus-negative inflammatory cardiomyopathy	IV methylprednisolone, IVIG	Systemic immunosuppressive agents are contraindicated during pregnancy. To be evaluated in the postpartum period, upon multidisciplinary team evaluation	(85, 86)

Treatment options for patients with PPCM and related arrhythmias are shown.

ACE, angiotensin converting enzyme; AF, atrial fibrillation; ARB, angiotensin receptor blockers; ARNI, angiotensin receptor neprilysin inhibitors; IV, intravenous; IVIG, intravenous immunoglobulins; LMWH, low molecular weight heparin; LQTS, long QT syndrome; LV, left ventricular; LVEF, left ventricular ejection fraction; LVNC, left ventricular noncompaction; MCS, mechanical circulatory support; MRA, mineralcorticoid receptor antagonists; PPCM, peripartum cardiomyopathy; S-ICD, subcutaneous implantable cardioverter defibrillator; SLE, systemic lupus erythematosus; UFH, unfractionated heparin; VKA, vitamin K antagonists; VT, ventricular tachycardia; WCD, wearable cardioverter defibrillator.

Among antiarrhythmic drugs, the use of amiodarone is restricted during pregnancy, since it can induce fetal hypothyroidism, growth retardation, and prematurity (73). Most betablockers, including sotalol, and central calcium-channel antagonists as verapamil, are well tolerated during pregnancy (60, 73). Instead, digoxin or flecainide should be used when benefits overwhelm risks (73–75), such as in the event of fetal arrhythmias (87). For unstable arrhythmias, electrical cardioversion is a suitable and safe option during pregnancy, but the presence of an obstetrician in advisable in light of the risk of increased intrauterine activity (11, 73). In our experience, betablocker and antiarrhythmic agents were employed in 63% and 44% of patients, respectively, without safety issues when used during pregnancy.

Among cardiac devices, pacemakers are indicated in case of severe bradycardia or CSD (78–80). Importantly, underlying arrhythmogenic diseases and/or risk factors for malignant ventricular arrhythmias should be carefully ruled out, to ensure that ICD are not needed instead. A guide for the clinical decision making is summarized in Figure 2. Given the transitory nature of PPCM and associated arrhythmias, WCD constitute a reasonable approach to protect pregnant women from arrhythmias in the short term. Women with a severe systolic dysfunction, as in PPCM with a LVEF under 35%, are more likely to manifest SCD from malignant ventricular arrhythmias (2, 8). Remarkably, the incidence of appropriate ICD shocks in PPCM was as high as 37% over a mean 3-year follow-up (88), i.e., at a significant longer term as compared to the postpartum period. Therefore, to avoid unnecessary ICD implant in primary prevention, WCD may be used for a few months with a bridge-to-recovery indication (2, 81, 82). Similar considerations are applied in the context of secondary prevention of SCD in patients presenting their first VT episode in pregnancy (4, 60). In this setting, withdrawal of WCD may be more challenging since no temporal cutoffs are available to notify the end-of-risk timing. It should be noted that, reflecting the local standard practice, no patients in our series had WCD. Consistently, in a recent consensus document of the Heart Rhythm Society (73), it has been reported that the criteria for early ICD placement should be more stringent compared to other cardiac conditions. This particularly applies to patients presenting with LVEF below 30% in conjunction with a LV end-diastolic diameter equal to or exceeding 60 mm, because of the low likelihood of LVRR even in the long term (89). The ESC guidelines recommend that for women presenting with symptoms and severe LV dysfunction 6 months after initial presentation, despite optimal medical therapy and left bundle branch block-shaped QRS with duration greater than 120 ms, cardiac resynchronization therapy should be strongly considered, because of the reported beneficial effects in classic PPCM (90). While transvenous devices may be placed even during pregnancy in selected cases, delivery prior to device implantation is advised for most PPCM patients (11). In fact, although the current reaching the fetus is minimal, transient fetal arrhythmias after electrical resynchronization have been described (91). Efforts should be also made to minimize fetal radiation exposure by limiting fluoroscopy and using abdominal shielding. Successful ICD implantation using echocardiography without fluoroscopy is a desirable option (92). Of 16 patients, the clinical indication to ICD implant applied to up to 10 patients (64%) by the end of follow-up.

Catheter ablation is another key therapeutic weapon that applies to a range of arrhythmias in a number of clinical scenarios. In the acute setting, catheter ablation is reserved for women suffering from hemodynamically unstable arrhythmias (10), as well as for VT persisting in spite of antiarrhythmic therapy (46, 47). In our series all patients received ablation late after clinical onset. As for cardiac device implant, also catheter ablation is preferred after delivery or when a pregnancy is planned in case arrhythmias have been already diagnosed (4, 46). This particularly applies to non-life threatening arrhythmias such as AF, as well as for reentry circuits likely to be completely abolished by ablation, such as WPW (73). Before performing catheter ablation in a pregnant woman, risk and benefit of both mother and fetus must be considered, because consequences include fetal radiation exposure, maternal hemodynamic imbalance, impaired placental perfusion (33).

As a final remark, among pathophysiology-directed strategies, the inhibition of prolactin secretion by means of bromocriptine in addition to standard heart failure therapy has shown promising results in two clinical trials (83, 84), but evidence is still contradictory (2, 8, 12), and no data are available about antiarrhythmic effects. Concerns have been raised also about drug-associated maternal adverse vascular events (93). For classic PPCM, the ESC included a weak recommendation (class II b, level of evidence B) for the use of bromocriptine (4). In our series on arrhythmic PPCM, only one patient (6%) received bromocriptine, but despite association with metoprolol she still required catheter ablation of PVC (Table 1). In selected patients with PPCM secondary to myocarditis, intravenous immunoglobulin administration has shown an improvement in LVEF (85). While no role is currently recognized for other etiology-driven therapies, it should be noted that three patients with EMB-proven virus-negative lymphocytic myocarditis underwent safe immunosuppressive therapy (86) in the postpartum period. All of them had uneventful follow-up, except for the need of PVC ablation in a patient with residual monomorphic PVC: results are consistent with the pleiotropic beneficial effects of immunosuppression in arrhythmic myocarditis (94), but it should deserve dedicated investigation as pathophysiology-guided therapy in the PPCM population. Our experience showed that 6 patients were ablated in the postpartum (38%), including 50% (2 of 4) of those showing LVRR during follow-up (Table 1).

**Figure 3:**
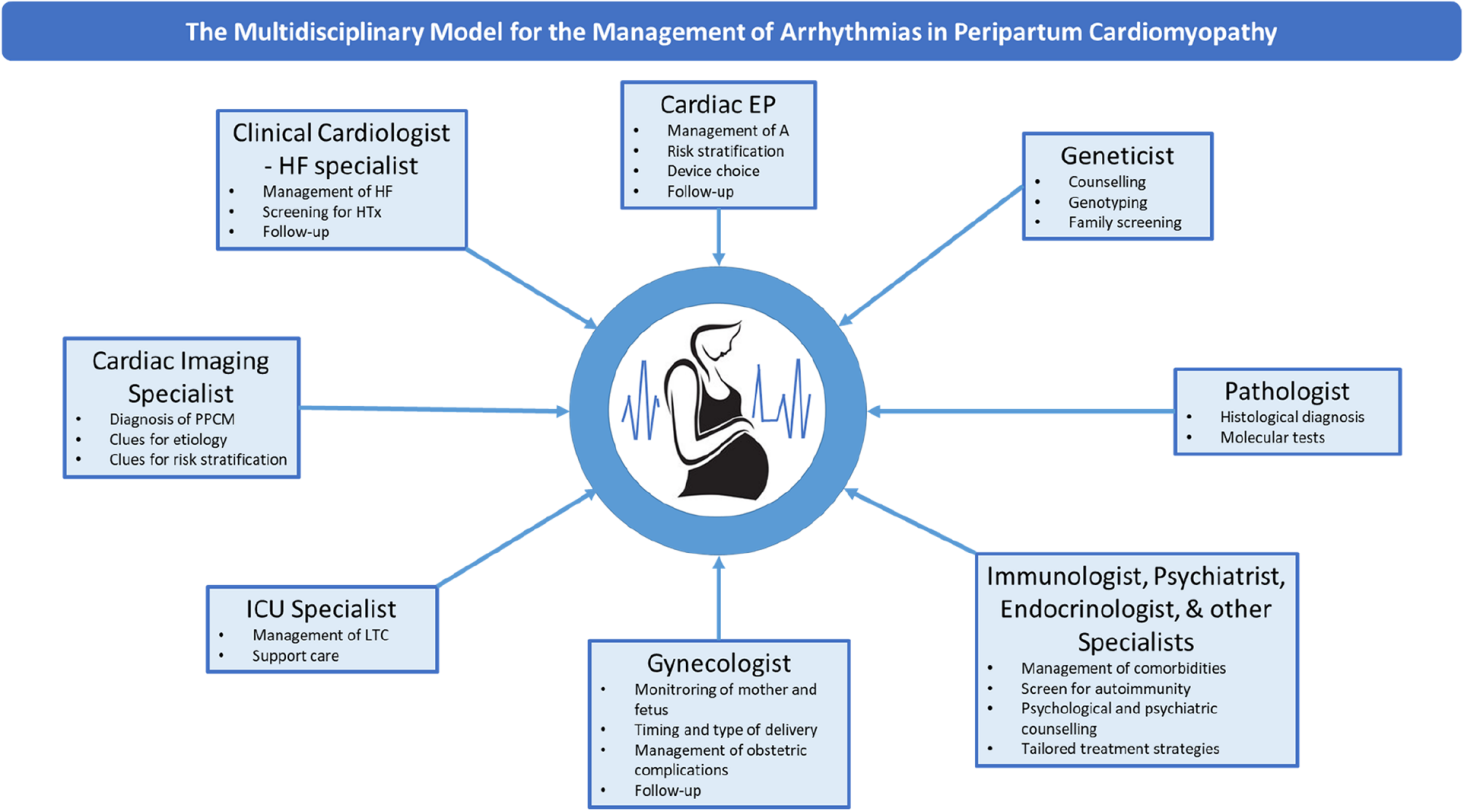
The multidisciplinary model for the management of arrhythmias in PPCM. The main components of the multidisciplinary healthcare team for the management of PPCM and related arrhythmias are shown. EP, electrophysiologist; HF, heart failure; HTx, heart transplant; LTC, life-threatening conditions; PPCM, peripartum cardiomyopathy.

Given the complex and multifactorial nature of the disease, multidisciplinary healthcare teams should become the gold-standard model of care, in compliance with the current recommendations applying to all cardiomyopathies (7). Figure 3 summarizes the model proposed based on the literature review and our own experience. On top of HF specialists, cardiac electrophysiologists have a critical role in decision making about management of arrhythmias and the prevention of SCD, both in the short and in the long term. Geneticists have a key contribution in defining clinical indications to genetic tests and enabling family screening. Gynecologists retain a key role, also for defining the mode and optimal timing of delivery. Other specialists may provide relevant contributions, such as immunologists and endocrinologists for the administration of pathophysiology-driven therapies. Also, since one patient in our series underwent arrhythmic SCD after refusing ICD and therapies, psychiatrists should assist in managing either preexisting or peripartum-associated mental comorbidities.

## Conclusions

PPCM is a complex and multifactorial disease, whose arrhythmic manifestations are currently under-investigated. While treatment choices are strongly conditioned by the pregnancy status, an open-minded and patient-tailored diagnostic workup is strongly encouraged to allow optimal treatment options after differential diagnosis is solved.

Efforts are needed to describe and further characterize the “arrhythmic” variant of PPCM, which posed hard clinical challenges for SCD risk assessment, as compared to the classic DCM phenotype with heart failure manifestations. In fact, differentiating bystander vs. PPCM-triggered arrhythmias, as well as revealing a missed preexisting diagnosis are a major issue, as shown in our case series. In these settings, multimodality diagnostic workup and multidisciplinary care models should be promoted. Similarly, regular follow-up is required in the long term to clarify the underlying diagnosis and prevent complications.

Given the association between PPCM and arrhythmic phenomena, which can even result in SCD, efforts are needed to early identify the best candidates to undergo definitive implantation of ICD. Multicentre prospective studies on well selected populations of PPCM patients are advocated, to substantially advance our knowledge in such a hot topic of modern medicine.

## Data Availability

The raw data supporting the conclusions of this article will be made available by the authors, without undue reservation.
